# Significantly increased levels of serum malonaldehyde in type 2 diabetics with myocardial infarction

**DOI:** 10.4103/0973-3930.60006

**Published:** 2010

**Authors:** Rashida Mahreen, M. Mohsin, Zahida Nasreen, M. Siraj, M. Ishaq

**Affiliations:** Bio Chemistry Department, Deccan Medical College, Hyderabad, India; 1 AMC, Bijapur, Karnataka, India; 2 Deccan Medical College, Hyderabad, India; 3 Osmania University, Hyderabad, India

**Keywords:** Malonaldehyde, Myocardial Infarction, Type 2 diabetes

## Abstract

**Aim/Objective:**

To see the levels of serum MDA in type 2 DM with MI. As Serum malonaldeyhde (MDA), a stable end product of lipid per oxidation produced by interaction of free radicals with membrane phospholipids, was estimated.

**Materials and Methods:**

30 type 2 diabetes mellitus cases with myocardial infarction and equal members of Type 2 diabetics without complications are enrolled. Thirty healthy subjects served as controls. Quantitative estimation of serum MDA levels were carried out in all the three groups, along with fasting blood glucose and total serum cholesterol.

**Results:**

It was found that serum MDA levels were significantly higher in both diabetic groups compare to the controls (*P*<0.01). Within the diabetic group, the serum MDA levels were significantly high in patients with myocardial infarction (*P*<0.01).

**Conclusion:**

The possible reason for significantly high serum MDA levels in cases of diabetic with myocardial infarction is due to tissue damage caused by myocardial infarction resulting in increased rate of production of free radicals as assessed by lipid per oxidation.

## Introduction

Hyperglycemia, the characteristic feature of diabetes, has been reported to be responsible for the elevated levels of free radicals in the plasma.[1]

There is recent evidence that increased oxidative stress in diabetes contributes to the development of diabetic complications.[2] Oxidation of lipids in plasma lipoprotein and cellular membranes is associated with the development of vascular disease in diabetes. Oxygen derived free radicals and reactive oxygen species interact with lipid bilayer of cell membrane resulting in lipid peroxidation.[3] Malonaldehyde (MDA) is a stable end product of lipid peroxidation.[4] A plethora of adverse physiological consequences of elevated MDA levels includes leakiness of cell membranes by altering structural integrity of membrane; inactivation of membrane bound enzymes, inactivation of surface receptor molecules leading to cell-regulating errors, the involvement of oxidized L.D.L in the foam cell formation leading to atherosclerosis has been documented. Although elevated levels of serum MDA have been reported in type 2 diabetes, there is paucity of literature on the extent of serum MDA levels in type 2 diabetes cases with myocardial infarction, a macrovascular complication.

Such studies may provide greater insight in the role of oxidative stress along with other predisposing factors in the precipitation of events like myocardial infarction and the need to use anti-oxidants as a prophylactic step.

## Materials and Methods

The study protocol was approved by the Institutional review board and all the subjects have given informed consent. Type 2 diabetics visiting the Outpatient Department of Medicine and Cardiology, Owaisi Hospital and Princess Esra Hospital, Hyderabad, were selected. They were divided in two groups.

Group I: Thirty patients of either sex diagnosed with Type 2 D.M without any complications. They were non-hypertensive, non-smokers, physical examination, X-ray Chest, E.C.G were done to rule out any chronic infections and ischemic heart disease. Estimation of serum creatinine levels, fundoscopy, and examination of deep and superficial reflexes, touch, position and vibration was done to rule out any complication.

Group II: Thirty patients of either sex having type 2 D.M with myocardial infarction. These subjects were confirmed type 2 D.M cases who had suffered an attack of myocardial infarction as confirmed by E.C.G changes. Blood samples were collected from them immediately after the attack.

Thirty patients having similar age groups of either sex comprised the control group; they were non-diabetic, non-hypertensive and non-smokers; 6 ml of venous blood was collected, 1 ml of blood in oxalate fluoride bottles for estimation of glucose, the rest in plain sterile bottles and allowed to clot. Serum was separated for estimation of MDA and total cholesterol. Estimation of MDA was done by thiobarbituric acid reactive substance assay according to the method described by Mahffouz.[5]

## Results

Details of the mean values along with the standard deviations for various parameters in the two diabetic groups as well as in the control are shown in the Table 1. The mean age was 50 ± 10.22 years in cases of type 2 diabetes without any complications and 53 ± 9.07 years in diabetic cases who had myocardial infarction. The control age group was 48 + 9.6 years.

**Table 1:** Results of various parameters in diabetic groups and diabetics with M.I. and control groups

Category	Mean age (Years) ± SD	Mean fasting blood glucose (mg% ± SD)	Total serum cholesterol (mg% ± SD)	Mean serum malonaldehyde (nmol/dl ± SD)
Control	48 ± 9.6	80.86 ± 7.56	193.13 ± 25.6	189.94 ± 33.64
Type 2 DM without complicatoins	50 ± 10.22	162.86 ± 35.57	292.56 ± 25.22	354.56 ± 76.44*
Type 2 DM with myocardial infarction	53 ± 9.7	180.43 ± 33.93	309.8 ± 31.94	547 ± 108.75*

P < 0.01 Control vs. type 2 DM without complications; Control vs. type 2 DM with myocardial infarction

Mean fasting blood glucose levels were 80.86 ± 7.56 mg% in control, 162.86 ± 35.57 in type 2 DM without any complications and 180.43 + 33.93 in the diabetes mellitus with myocardial infarction Statistically analysis revealed that mean FBG level in both types of DM were significantly higher (*P* < 0.05) than that in the control group, however when the mean FBG value in type 2 DM without any complications was compared with those with myocardial infarction, no significant differences were observed.

Serum total cholesterol was estimated in the test as well as in control groups as this is one of the important parameters which may be correlated to myocardial infarction. Mean total cholesterol levels were 193.13 ± 25.6 mg% in the control group and 292.56 ± 25.22 in the diabetic without complications and 309.8 ± 31.91 mg% in type 2 DM with myocardial infarction. The differences between test groups and control were statistically significant (*P* < 0.05).

The mean serum MDA levels in diabetics with myocardial infarction were highest 547 ± 108.75 nmol/ (dl) followed by 354.56 ± 76.44 nmol/(dl) in diabetics groups without any complications. In the control groups, MDA was found to be 189.94 + 33.64. In both test-groups the serum MDA level were significantly higher compared with the controls (*P* < 0.01) and in between the diabetic groups it was found to be significantly higher in diabetes with myocardial infarction (*P* < 0.01).

## Discussion

It is well documented that MDA is a stable end product of free radicals induced by lipid peroxidation. Thus MDA serves as a reliable marker for the assessment of free radical induced damage to tissues. In diabetic patients a major factor that is responsible for enhanced free radical generation is hyperglycemia through auto-oxidation of glucose; it may be an important risk factor for cardiovascular disease.[6]

In both groups of type 2 D.M, serum MDA levels were significantly higher than the normal. In type 2 DM with myocardial infarction, MDA levels were significantly higher than Type 2 cases without any complications. This may be explained on the basis of longer duration of the disease leading to excessive free radical induced damage, another reason for the high levels of MDA could be the glycation of serum proteins leading to activation of receptors for advanced glycation end products (A.G.E), which initiates the process of atherosclerosis.[7] Excessive oxygen derived free radicals are generated in the early stage of myocardial infarction.

Involvement of oxygen free radicals in the pathophysiology of inflammation ischemia and reperfusion damage in a number of organs and tissues had been reported in literature.[8]

In type 2 diabetics without complications similar free radical induced damage may be in progress, which may require further period of time to manifest as macro-vascular complication. Oxidative stress may inhibit NO mediated endothelial function by degrading NO.[9] It has been seen that high levels of lipid peroxides reflect increased oxidative stress; this may cause decrease circulating anti-oxidants due to increased consumption.[10]

Apart from good glycemic control, supplementation by anti-oxidants in subjects of increased MDM levels in type 2 DM may help in reducing oxidative stress which is one of the causes of myocardial infarction.
